# Antibiotic stewardship through clinical data digitization: perceived opportunities and obstructions by medical doctors from semi-urban setting in central India

**DOI:** 10.3389/fdgth.2025.1652086

**Published:** 2025-09-10

**Authors:** Mala Kanthali, Gautam Bhagwat, Ashish Pathak, Manju Purohit

**Affiliations:** ^1^ Department of Pathology, R.D. Gardi Medical College, Ujjain, India; ^2^ Department of Pediatrics, R.D. Gardi Medical College, Ujjain, India; ^3^ Department of Global Public Health, Health Systems and Policy (HSP): Medicines Focusing Antibiotics, Karolinska Institutet, Stockholm, Sweden

**Keywords:** antibiotic, stewardship, resistance, digitization, electronic health records, qualitative research

## Abstract

**Background:**

Antibiotic resistance is a global public health concern. Inadequate record-keeping and irrational antibiotic prescriptions are challenging factors for antibiotic stewardship. This study explores the perceptions of medical doctors in a semi-urban setting in India, regarding the role of clinical data digitization in mitigating antibiotic resistance.

**Methods:**

The study was conducted at R D Gardi Medical College located in a semi-urban district of Central India. Qualitative data from 20 medical doctors from government and private sector were collected through in-depth, semi-structured interviews of which 18 interviews were analyzed using thematic analysis following Braun and Clarke's framework.

**Results:**

Two major themes emerged from four overarching subthemes: (1) digitization enhances accountability and continuity of care, (2) potential for local antimicrobial surveillance, (3) infrastructural and technological barriers to adoption, and (4) the necessity of government support and capacity building. The participants believe that digitization could help in rational antibiotic prescription if there is a government mandate and infrastructure feasibility in resource constrained settings.

**Conclusion:**

Clinicians in semi-urban India perceive the digitization of clinical data as a promising tool to combat antibiotic resistance. However, systemic and infrastructural challenges must be addressed to utilize its full potential.

## Introduction

Antibiotic resistance is recognized as a critical global health issue. The World Health Organization (WHO) has categorized antimicrobial resistance as one of the top ten global public health threats of the 21st century (1). India has been identified as one of the world's largest consumers of antibiotics and a hotspot for resistant pathogens (2). The easy accessibility of over-the-counter antibiotics without prescriptions, along with underutilization of diagnostic tools in clinical practice, particularly in rural and semi-urban settings, has led to empirical rather than evidence-based decisions (3). This overuse and misuse of antibiotics are compounded by poor documentation of patient outcomes by inadequate surveillance systems and complex healthcare delivery (4–6). This poses a severe risk not only at the patients’ level but also at the community level as resistant strains may spread across communities and the environment.

The digitization of clinical data has emerged as a promising tool to support antimicrobial stewardship and improve health outcomes. Electronic health records can enhance diagnostic accuracy, support clinical decision-making, and facilitate longitudinal tracking of patient histories and prescription patterns (7). Digitized health information systems have been instrumental in generating real-time resistance data, promoting judicious antibiotic use, and enabling public health authorities to mount coordinated responses (8). However, these advances remain unevenly distributed. While tertiary care hospitals and corporate healthcare chains in India have adopted digital platforms, semi-urban and rural settings often lag due to infrastructural, logistical, and training-related barriers (9). The National Digital Health Mission (NDHM), launched in 2020, aims to bridge this digital gap by promoting a uniform use of electronic health records even in rural and semi-rural settings (10). Yet, the success of such initiatives is hindered not only on the implementation policy level but also on the ground-level receptivity and capacity of medical practitioners to use it routinely for patient care, antibiotic prescription and stewardship.

Considering the role of healthcare providers in combating antibiotic resistance, it is necessary to examine their views and practices on clinical data digitization in semi-urban and rural Indian settings. While existing literature has primarily focused on technical feasibility and policy framework concerns for digital health systems, with some attention to the subjective experiences and concerns of physicians (11–15), understanding how these professionals perceive the potential of digitized clinical data in reducing antibiotic resistance is crucial for developing interventions that are both contextually appropriate and practically implementable (16). This study aims to fill this gap by investigating the perspectives of medical doctors in a semi-urban district of Central India on the utility, feasibility, and challenges of using digitized clinical data to combat antibiotic resistance. The research involves qualitative interviews with doctors both from the government and private sectors.

## Materials and methods

### Study design

We conducted a qualitative study using semi-structured interviews to explore clinician's perspectives on the use of digital health records and antimicrobial stewardship practices in both public and private healthcare settings. The study adheres to the Consolidated Criteria for Reporting Qualitative Research (COREQ) 32-item checklist to ensure transparency and rigor in reporting the research process (17).

### Study setting

The data was collected and analysed by the Central Research Laboratory, R D Gardi Medical College, Ujjain district, Madhya Pradesh. The data was collected from February 2023 to June 2023 from the Ujjain district which represents a mix of urban, semi-urban and rural healthcare services with a dual health system comprising government-run primary health centers (PHCs), community health centers (CHCs), and district hospitals, alongside a large sector of private clinics, hospitals and solo practitioners, representing most of the health care set up in India. The population has access to tertiary care centers, with patients often relying on outpatient services at neighbourhood private clinics or local government facilities for primary and secondary healthcare needs. Some corporate hospitals in the district have digital health tools and software, which are not used for antibiotic prescription decisions or antibiotic stewardship. The majority of patient records are paper-based. There is no uniform electronic health record system, though fragmented data for infectious diseases are in place at the district level, real-time access to antibiotic susceptibility and antibiotic use data is limited or absent. Healthcare providers frequently encounter patients with infectious diseases such as respiratory tract infections, diarrheal illnesses, urinary tract infections, and skin conditions for which antibiotics are frequently prescribed (3, 18).

### Participants

Total 50 doctors with variation in medical backgrounds and experiences, such as general practitioners, physicians, surgeons, and obstetricians with either basic medical degrees or postgraduate qualifications were identified as eligible for participation based on predefined criteria. The inclusion criteria were: (1) currently practicing as a registered allopathic medical doctor in Ujjain District, (2) a minimum of five years of clinical experience and (3) regular involvement in patient care where antibiotics are commonly prescribed. Efforts were made to include a diverse sample in terms of age, gender, years of experience, sector of practice (government vs. private), and practice setting (urban vs. peri-urban or rural) (Table 1) to capture a broad range of perspectives reflective of the heterogeneous landscape of clinical practice in semi-urban India. Participants were excluded if they: (1) were retired or not currently practicing, or (2) declined to provide informed consent. From the identified 50 eligible participants, 20 doctors consented to participate in the study and were scheduled for in-depth interviews.

**Table 1:** Participant demographics and experience with digital tools.

Participant ID	Gender	Age	Practice sector	Speciality	Primary practice setting	Years of experience	Digital tool experience	EMR familiarity
P1	Male	34	Government	Physician	Primary health centre	8	Moderate	Yes
P2	Female	46	Government	Obstetrician	Community health centre	20	Low	No
P3	Male	38	Private	Surgeon	Urban private clinic	8	High	Yes
P4	Female	29	Private	General Physician	Semi-urban general practice	5	High	Yes
P5	Male	50	Government	Paediatrics	Community health centre	23	Low	Yes
P6	Female	33	Private	Surgeon	Rural private dispensary	7	Moderate	Yes
P7	Male	40	Private	Physician	General urban clinic	10	High	Yes
P8	Female	37	Private	Obstetrician	Solo clinic	10	Moderate	Yes
P9	Male	55	Government	Surgeon	District hospital	28	Moderate	Yes
P10	Male	60	Private	Physician	Urban clinic	32	Low	Yes
P11	Female	45	Government	Obstetrician	Maternal and child health unit	15	Low	Yes
P12	Male	36	Private	Paediatrics	Family medicine	07	Moderate	Yes
P13	Male	31	Private	Physician	Urban solo clinic	5	High	Yes
P14	Female	61	Private	Orthopaedic surgeon	Urban solo clinic	30	Moderate	Yes
P15	Female	37	Government	Obstetricits	Primary health centre	8	Moderate	Yes
P16	Female	40	Government	Obstetrician	District hospital	9	High	Yes
P17	Male	51	Government	Orthopacian surgeon	District hospital	22	High	Yes
P18	Male	62	Private	Physician	Rural solo clinic	32	Moderate	Yes

### Data collection

Data was collected using a semi-structured, open-ended introductory probing topic guide (Supplementary Information S1) developed through an extensive review of the literature on antibiotic resistance, digital health adoption, and health systems in low-and middle-income settings (19, 20). The guide was refined based on input from public health researchers, infectious disease specialists, and qualitative methodology experts. Core topics included: patterns of antibiotic prescribing in outpatient settings; methods of medical record-keeping (paper-based, digital, hybrid); familiarity with and use of digital health tools (e.g., mobile applications, electronic health records); perceived facilitators and barriers to clinical data digitization; the potential of digitization to influence prescribing behaviour and resistance patterns; and recommendations for digital health policy and implementation in rural settings.

The interviews were conducted in person by MK depending on the availability and time preferences of the participants. The interviewer was trained in qualitative interviewing. Informed consent was obtained from the participants before the interview after explaining the research objectives. Participants were assured the opportunity to leave the interview or ask any question during the interview sessions. Interviews were conducted and audio-recorded mainly in Hindi or English or a mix of both languages and lasted between 25 and 45 min. The researcher kept a journal while collecting and recording the data to reduce potential interviewer bias. All interviews were transcribed verbatim. Hindi transcripts were translated into English by bilingual researchers experienced in healthcare terminology. The translations were reviewed by a second researcher to ensure semantic accuracy and preservation of meaning on the same day or as soon as possible. Any discrepancies were resolved through consensus. Participant names and identifying details were removed or anonymized to ensure confidentiality.

### Data analysis

We employed inductive content analysis to explore patterns and emergent themes from the interview data. The analysis followed Braun and Clarke's (2006) six-phase approach, beginning with repeated reading of transcripts to achieve data familiarization. The analysis consisted of identifying meaning units, condensing it and assigning codes to identical condensed units. Line-by-line open codes were generated and organized manually and independently by two researchers to ensure comprehensive engagement with the text. These codes were then reviewed to identify similarities and differences, and grouped into tentative categories. Categories were then organized, leading to the identification of sub-themes under a theme. The process was repetitive, with codes and themes refined through multiple rounds of discussion and constant comparison among authors. Discrepancies between coders were resolved through consensus. Data collection and analysis continued until no new codes or themes emerged from the data. Data saturation was reached after 16 interviews, however, to confirm saturation, additional interviews continued, which reiterated previously identified patterns without contributing new conceptual insights.

To ensure trustworthiness and rigor, we adhered to the criteria established by Lincoln and Guba (1985): credibility, transferability, dependability, and confirmability. Credibility was enhanced through investigator triangulation and peer debriefing. An audit trail was maintained to document analytical decisions and theme development. Special attention was given to accurately represent participants’ views in the final themes, ensuring that findings were grounded in the data.

### Ethical considerations

The study protocol was reviewed and approved by the Institutional Ethics Committee of the institute (20/2022). All participants were provided with written and verbal information about the study's objectives, methods, and confidentiality protocols. Written informed consent was obtained from each participant prior to data collection. Participation was entirely voluntary, and participants were informed of their right to withdraw at any time without any consequences. To protect the participants’ privacy, all transcripts were de-identified using pseudonyms, and any contextual details that could potentially reveal the identity of the participant were omitted or modified. Audio recordings, transcripts, and coded data were stored on password-protected devices accessible only to the research team.

## Results

### Participant characteristics

Of the twenty clinicians recruited, eighteen interviews were included in the final analysis. Two interviews were excluded from the analysis due to incomplete data for several core questions. The interview sessions were prematurely terminated owing to an unforeseen interruption, and follow-up attempts to reschedule were unsuccessful. Among the eighteen interviews analyzed, eight participants were female and ten were male, with ages ranging from29 to 62 years (Table 1). There was a balanced representation from both public and private healthcare sectors. Participants held in diverse roles, including general outpatient consultations, maternal and child health, surgery and management of infectious diseases such including tuberculosis. They are from various clinical environments—from government primary health centers to solo private clinics. This diversity in experience and settings provided a comprehensive perspective for the study's objective. The mean duration of clinical practice was 15.5 years (range: 5–32 years).

Two main themes were presented here incorporating emergent findings which were1. **Digitization for accountable, informed, and coordinated antibiotic stewardship, 2. Contextual Barriers to clinical data digitization in semi-urban healthcare Settings**. Four subthemes have emerged of the categories reflecting key perceptions, supported by direct quotations that exemplify participant views mentioned below in italics. Table 2 provides the domains and subthemes identified, aligned with the categories and codes derived from participant narratives.

**Table 2:** Framework matrix of digitization of clinical data for antibiotic stewardship from the study conducted among medical doctors in ujjain, central India.

Theme	Sub-theme	Categories	Codes	Illustrative Quote
Digitization for Accountable, Informed, and Coordinated Antibiotic Stewardship	Digitization enhances accountability and continuity of care	Avoiding unnecessary prescriptions	Lack of access to previous prescriptions; duplication risk	“We often don't know what antibiotics were given before. Electronic medical records would reduce overlap.” (P3)
		Better follow-up, and support	Memory aid for clinical continuity; helps during re-visits	“A digital record helps remember where we left off during the last visit.” (P10)
		Communication between providers	Absence of referral records; fragmented care between providers	“If patient shifts to another clinic, they carry no files. A centralized system helps.” (P4)
	Potential for Localized Resistance Surveillance	Need for local data	Geographic variation in resistance patterns	“Each region has different bugs. We need local data, not national averages.” (P5)
		Digital integration with laboratories	Linking lab reports to clinical systems; real-time surveillance	“If labs upload results, we can see patterns—what's working, what's not.” (P7)
		Decision-support potential	Algorithmic suggestions based on antimicrobial susceptibility	“Now a days software could even suggest likely effective antibiotics based on resistance data.” (P7)
Contextual Barriers to Clinical Data Digitization in Semi-urban Healthcare Settings	Infrastructural and Technological Barriers	Poor connectivity	Unstable internet limits real-time access	“Internet is unreliable here. It can't be the backbone of the system.” (P5)
		Lack of equipment	Clinics lack digital infrastructure (computers, EMRs)	“We have no desktop or laptop. We do everything by hand.” (P1)
		Digital skills gap	Low digital literacy, especially among senior clinicians	“Many older doctors here don't even use Smartphone properly.” (P11)
		Cost and maintenance	Financial burden of implementing and sustaining digital systems	“Installing and maintaining systems is expensive for a small clinic.” (P8)
	Necessity of Government Support and Capacity Building	Policy mandates	Need for government-led mandates to ensure adoption	“Unless the government mandates and monitors this, adoption will be minimal.” (P10)
		Financial incentives	Incentives as motivators for private and small providers	“If digital adoption comes with incentives, clinics will participate.” (P6)
		Training programs	Training and skill-building required for meaningful system usage	“Training is crucial. Without it, systems will be underutilized.” (P5)

### Subtheme 1: digitization enhances accountability and continuity of care

#### Category 1.1: Avoid unnecessary prescribing

Clinicians emphasized that the electronic medical record could play a critical role in preventing duplicate prescribing of antibiotics, especially in cases where patients see multiple providers. Currently, clinicians rely mostly on patient reminders or paper prescriptions, which are often incomplete or lost. This lack of coordinated data sharing leads to repeated administration of the same or multiple ineffective antibiotics. Access to full treatment histories through electronic medical records will enable more rational and informed prescribing practices.

“We often don’t know which antibiotics have been administered previously. If a patient turns up with no previous records, it’s like starting from scratch. A digital record system gives us a complete picture immediately…Electronic records would reduce overlaps……….” (P3)

“At the moment we mainly rely on memory or paper notes…sometimes patients forget what they have taken or get names mixed up. Digital records help us to track which antibiotics have been administered and avoid overlaps…saves us the guesswork.” (P4)

In addition, several doctors pointed out that digital systems could encourage more judicious use of antibiotics by holding prescribers accountable through data trails.

“If we know our prescriptions are being recorded and reviewed, we will think twice before casually administering antibiotics…and make our decisions more responsible.”(P6)

#### Category 1.2: better follow-up and support digital records

Were seen as helpful to improve patient follow-up and continuity of care, especially in high-volume clinics. Many participants described scenarios where patients did not adhere to treatment plans or lost previous prescriptions, making subsequent consultations difficult. Participants emphasized that electronic medical records connected to the internet could ensure a longitudinal view of patient history and reduce fragmented care, especially for chronic infections such as tuberculosis or COPD.

“A digital record helps us remember where we left off at the last visit.”(P3)

“If we had a system where previous treatment was available digitally… it would reduce abuse, especially for diseases with chronic treatment such as tuberculosis or COPD.”(P10)

“It’s frustrating when you find out that a patient was given the same antibiotic last week. That’s the kind of error that electronic records could prevent.” (P12)

#### Category 1.3: communication between care providers

Clinicians emphasized the facilitation of communication between care providers through a digitized central data system. In semi-urban areas, where patients frequently move between the public and private sectors or from one clinic to another, continuity of care is maintained thanks to accessible medical records. A centralized digital system would enable the seamless exchange of information, avoid repeat examinations and improve coordinated care across facilities. Both government and private sector participants concede that in the absence of a recorded treatment history, doctors typically over-treat to be safer for the patient, and also for reasons of social desirability.

“If a patient transfers to another clinic, they don't have any records with them. A centralized system helps.” (P4)

“At the moment, everything is handwritten. We often don't know what the patient has received from another doctor…Continuity of care is interrupted if each doctor works in isolation. Shared records couldfix that…………….electronic records can really help us coordinate better.”(P3)

### Subtheme 2: potential for localized real-time antimicrobial resistance surveillance

#### Category 2.1: need for local data

Clinicians emphasized the need for antimicrobial resistance data and antibiotic guidelines specific to their geographical region rather than working on national guidelines which may not reflect the local resistance patterns. The discrepancy between guideline-based therapy and field reality leads to ineffective treatment regimens for patient's illness.

“Each region has different bugs. We need local data, not national averages…….if we have shared database showing local resistance patterns, our treatments would be more effective.” (P5)

#### Category 2.2: digital integration with laboratories and pharmacies

Many clinicians suggested that the integration of laboratory diagnostic systems with clinical records would allow real-time access to susceptibility reports from the microbiology laboratory. Currently, timely adjustment of antibiotic regimens is hampered by the delay in receiving laboratory results or the lack of coordination between diagnostic and clinical teams. Digital integration was seen as a way to create antibiograms and make treatment decisions more effectively.

“When the labs upload their results, we can see patterns — what works, what doesn't…any times the reports don't reach the point of care in time…if we had a networked system…the reports could be viewed from our system at the point of care or with our mobile app in my hand, even if I'm not in the hospital…and give me the ability to update my treatment based on them……….."(P7)

“With point-of-care test results, we could stop antibiotics earlier……., but without this; we play it safe and sometimes over-prescribe…we really need systems that talk to each other. If the lab, pharmacy and patient records are connected, we can identify patterns early and make better treatment decisions…we could prescribe better…right now we're just guessing.”(P14)

#### Category 2.3: potential for decision support

Clinicians also envisioned the use of advanced software tools that could provide clinical decision support based on real-time resistance data. These tools could help with antibiotic selection and highlight potential discrepancies between the diagnosis and the prescribed antibiotic. Such software could significantly reduce irrational prescribing.

“Today, there is a variety of software that can even suggest likely effective antibiotics based on resistance data…these digital tools are designed to work seamlessly with electronic records so that clinicians can access resistance data and patient history in real time to make informed decisions…..”(P7)

“……..Data guides antibiotic prescribing…By analyzing real-time resistance patterns and patient needs, this software…can… also eventually recommend the most effective treatment options…The ability to incorporate local resistance trends into prescribing decisions can ensure that clinicians are not relying on general guidelines, but are making the right choice for their specific patient……."(P13)

### Subtheme 3: infrastructural and technological barriers

#### Category 3.1: poor connectivity

Issues, especially in rural areas, emerged as one of the primary obstacles to digitization. Without reliable internet access, clinicians felt that online electronic medical records and laboratory systems would have limited use and would not be ideal or practically sustainable. This limitation emphasizes the need for offline systems that can be synchronized when the internet or electricity connectivity is restored.

“Internet is unreliable here. It can’t be the backbone of the system.”(P5)

“We don’t have the basics—no Wi-Fi, sometimes no power. How can we run digital systems?…………electricity is not consistent here, forget about internet”(P2,11)

#### Category 3.2: lack of equipment

Several participants highlighted the absence of basic digital infrastructure in their clinics. Lack of desktops, printers, and even Smart-phone were identified as barriers to electronic records implementation.

“We have no desktop or laptop. We do everything by hand.”(P1)

“Without active surveillance, resistance spreads silently. We need live dashboards showing resistance hotspots in the hospital."(P9)

“Look at the urban health centres—they have tablets, apps, even dashboards. If the same is given here, we can use it too.” (P6)

#### Category 3.3: lack of digital skills

Was cited as one of the biggest limitations. Many were not even familiar with using basic software tools, which hindered the implementation of a digital system.

“Many of us…doctors here can’t even use smart phones properly.”(P11)

Training and ease of use were cited as important design principles for any new digital tool.

“Most software is designed for large hospitals. In our environment, it’s too slow or too complicated…a simple digital platform…like in other government programs … it should be operated with a mobile phone…to enter new antibiotic prescriptions…we would need mobile-friendly systems."(P10, P15)

#### Category 3.4: costs and maintenance

Concerns about the financial costs of implementing and maintaining digital systems were particularly pronounced among small private clinics. Without external support, most did not feel able to support the infrastructure and recurring expenses. The need for scalable, cost-effective solutions such as mobile-based Electronic medical records was emphasized by many.

“Installing and maintaining systems is expensive for a small clinic.” (P8)

### Subtheme 4: necessity of government support and capacity building

#### Category 4.1: policy mandates

Participants emphasized that voluntary adoption of digital systems would not reach critical mass without policy mandates. Government-led initiatives, similar to those used in the national programs, were seen as essential for standardizing practices.

“Unless the government mandates and monitors this, adoption will be minimal.”(P10)

“If the government launches and monitors the digital platform for diagnosis, name of test……….antibiotic prescription…….as the digital platform in the tuberculosis programme, ………. the generation of patient ID at any centre can be seen anywhere to track past diagnosis…… and treatment……….can be seen from mobile handset”(P15)

“the strict policy for antibiotic prescription should be there…………the generation of antibiotic prescription for pharmacy should always be….from the digital platform……strict monitoring of the pharmacy to sell antibiotic only on prescription………kind of prescription having some government code or mark”(P18)

#### Category 4.2: financial incentives

Several clinicians noted that financial incentives, such as subsidies or performance-linked payments, could accelerate adoption, particularly for the private sector.

“If digital adoption comes with incentives, clinics will participate.”(P6)

“Without incentives or mandates from the government, most private practitioners won't adopt digital systems.” (P14)

#### Category 4.3: training programs

The role of regular training programs was emphasized across the board. Participants stated that without structured education and ongoing support, even the best systems would be underutilized.

“Training is crucial. Without it, systems will be underutilized.”(P5)

“Unless the government pushes it and provides training, people won’t adopt it. We’re already overloaded."(P14)

“Hospitals are focusing on educating healthcare professionals to ensure smooth adoption. Training programs help clinicians understand how to interpret data-generated recommendations and integrate them into their workflow……………….” (P12).

## Discussion

**Figure 1:**
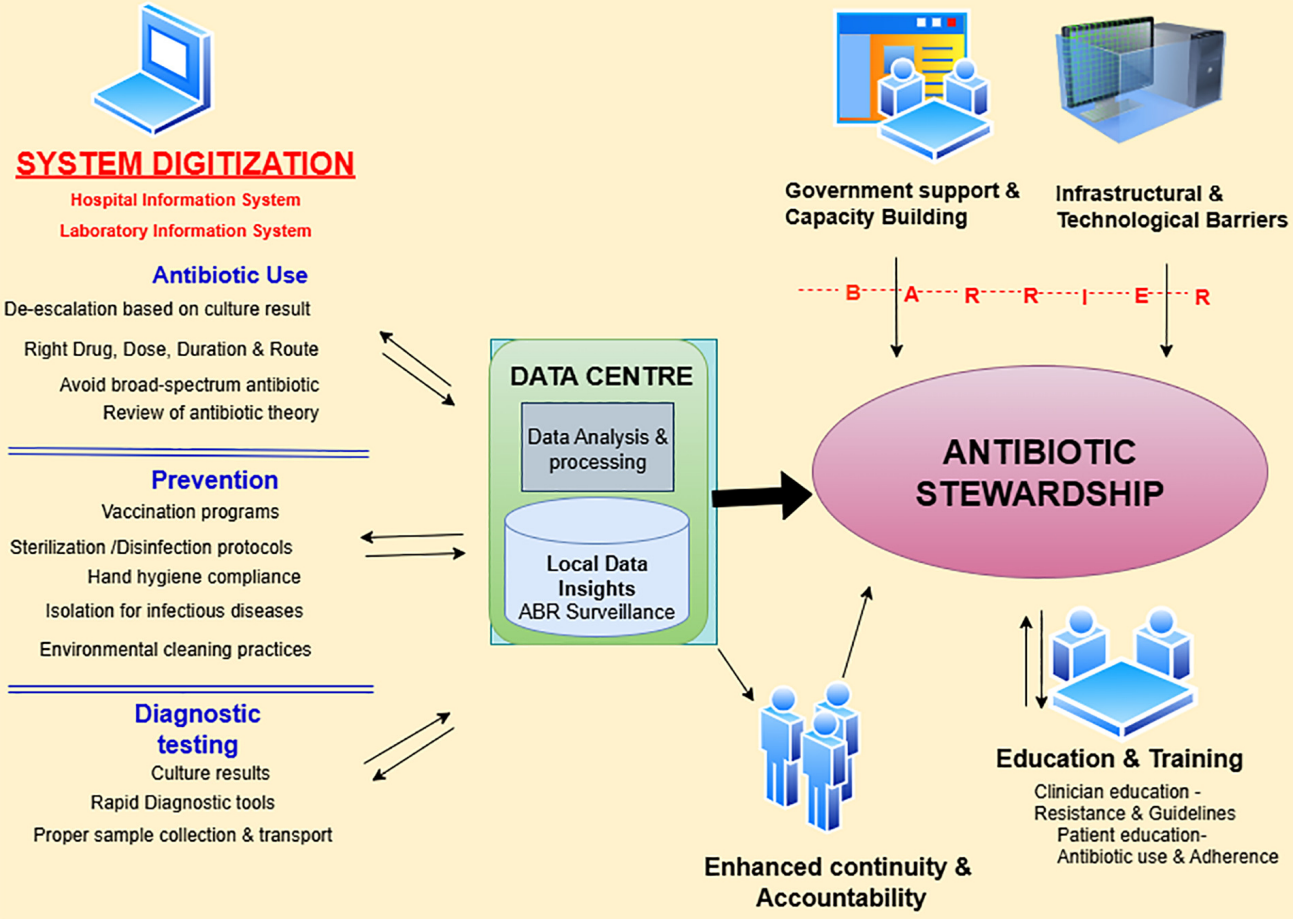
Conceptual dynamics for effective antibiotic stewardship through clinical data digitization in healthcare settings based on the findings of the study.

We examined thoughtful view of exploiting clinical data digitization for antibiotic stewardship in semi-urban and urban areas of India.We discovered that digital systems can be strategically implemented to address antibiotic resistance within India's diverse healthcare infrastructure and can be incorporated within existing digital health care platforms/systems (21). While the potential benefits for antibiotic stewardship, care continuity, and local surveillance were well recognized, infrastructural challenges and the lack of policy support were also preventive factors for implementing digital solutions for antibiotic stewardship (Figure 1).

The study reveals that clinicians recognize the transformative potential of electronic medical records in reducing antibiotic prescriptions. Many clinicians (12/18) reported that, in the current scenario, treatment history is often reconstructed based on patient recall or physical prescriptions, which are incomplete or lost, leading to poly-pharmacy or repeated irrational antibiotic use— a known risk factor for antimicrobial resistance (22, 23). By enabling access to accurate and longitudinal patient histories, electronic records could foster more rational prescribing practices. Moreover, digital records are critical for improving follow-up and bridging care transitions between providers, reducing treatment gaps and inappropriate empirical therapies (24, 25). Clinical data digitization will enhance data accuracy, transparency, and reduces human error, which is crucial for controlling antibiotic misuse (16, 25–27). The observed enthusiasm for provider-to-provider communication through a centralized health information system is not just a logistical aid but can also be a mechanism to strengthen prescriber responsibility in a setting where patients frequently “clinic-hop” across the public/private sector or informal health providers (IHPs) (often without formal medical degrees—serve as the first point of contact) in rural India. We have previously shown that IHPs frequently prescribe antibiotics empirically (3, 18), sometimes inappropriately, thus contribute significantly to antimicrobial resistance trends.

The digital integration between laboratories, pharmacies, and clinical records is one of the key enablers for timely recognition of antibiotic resistance trends. In India, where the antimicrobial resistance is compounded by over-the-counter antibiotic use, lack of diagnostic stewardship, and suboptimal record-keeping, a synchronized hospital-and laboratory-information-systems can act as a foundational pillar for the systemic change (24, 28). The time-sensitive access to laboratory results can significantly reduce inappropriate empiric antibiotic use, shorten the time to effective therapy, and prompt de-escalation or modification of antimicrobial therapy—all essential for successful antimicrobial stewardship (29–32). Furthermore, the electronic medical records support real-time data exchange between the microbiological diagnosis and clinical intervention, generating the localized antibiotic resistance surveillance system. Studies have shown the importance of real-time surveillance systems in reducing antibiotic resistance (7, 16, 25, 26, 31). Antibiograms, generated from patient digital records, could assist healthcare providers in selecting the most effective antibiotics and developing institutional antibiotic policy (32). The emerging artificial intelligence (AI)-based clinical decision support systems (CDSS) could help bridge the gap by supporting frontline providers with evidence-based recommendations (33) if these AI tools are context-sensitive and trained on local clinical data to ensure relevance and accuracy. Also, as pointed out by participants, when practitioners know that antibiotic prescriptions are being monitored digitally, they are more likely to act judiciously as studies have emphasized the potential of these AI surveillance-linked accountability leads to improved prescribing practice and care (34) by flagging inappropriate prescriptions (31). In India, where resistance to common antibiotics is widespread, such surveillance systems could be a valuable asset in improving clinical outcomes and controlling antibiotic resistance.

Despite recognizing the potential benefits, infrastructural deficiencies hinder the adoption of digital systems in routine practice (35, 36). Intermittent electricity supply, unreliable internet connectivity, lack of access to digital hardware such as computers or tablets, budgetary allocation for digital transitions, and lack of trained personnel to manage digital systems and institutional support are challenges in real-life situation. Concerns regarding interoperability, data loss, data standardization, maintenance of digital tools and fear of litigation were also cited as reasons for limited enthusiasm toward digitization without parallel improvements in infrastructure and system design. The additional time required to input digital data also limits transitioning from paper records to digital adoption, especially in high-volume settings in both the private and government sector (37).

Our findings emphasize the importance of contextually appropriate digital solutions that can operate in offline modes, reduce the workload for healthcare providers, and be tailored to local infrastructure limitations. Voluntary digitization alone would not reach critical mass without support from government policies, mandates and institutional incentives (38). In India, the government has recognised the value of aggregated data in identifying the local resistance trends. Initiatives, like the National Antibiotic Resistance Surveillance Program (NARSP), have made progress by implementing surveillance systems (39–41). Integrating AI-CDSS with national digital health strategies holds substantial potential by its successful deployment in rural and under-resourced settings and phased implementation. The Ayushman Bharat Digital Mission (ABDM), has shown that government leadership is crucial for scaling digital health solutions in India (42). Policy frameworks under India's NDHM provide an environment for integrating hospital and laboratory information system, and electronic medical records (43).

Drawing from India's experience with digitized tuberculosis management, similar frameworks could be extended to contain antibiotic resistance. Incentivizing the use of digital health tools such as mobile-first platforms and e-health platforms through capacity-building, training, reduced workload, performance-linked funding and recognition programs for clinics and healthcare providers actively engage in antimicrobial stewardship through digitization could accelerate this transformation. To address digital competency gaps and ensure sustainability, training programs should be integrated into broader public health system strengthening efforts. Improving user-friendly mobile-based systems or applications that function even in the absence of electricity or internet facilities, offer local language support, and are not time-intensive are crucial to enhance physicians’ attitudes towards digital systems (44). Public-private partnerships could also accelerate digital health infrastructure, especially in resource-constrained settings where government clinics alone may not have the capacity.

While our qualitative study provided a comprehensive understanding of how digitization of clinical data can impact antibiotic stewardship in a semi-urban Indian setting,it has some limitations. The sample size of 18 participants, while adequate for qualitative analysis,limits the generalizability of the findings. Further studies with larger, more diverse sample encompassing a broader range of healthcare providers, including nurses, pharmacists, and public health officials are needed to gain a more comprehensive understanding of digitization in antibiotic resistance control. Reliance on self-reported data may introduce social desirability bias and researcher bias in interpreting meaning cannot be entirely eliminated, despite interview coding. Conducting the interviews mostly in clinical settings may have influenced the openness of responses and time constraints might have limited fuller exploration of certain views. Finally, while the study was conducted in a semi-urban district, the findings may not be applicable to more urban or remote rural areas, with differing the healthcare infrastructure and challenges.

In conclusion, the findings of our study emphasize that clinical data digitization is a promising tool for antibiotic stewardship in semi-urban India. While healthcare providers recognize the potential benefits, particularly in improving accountability, care continuity, and localized resistance surveillance, significant barriers, such as infrastructure limitations and user-friendly technological challenges must be addressed. Policy-level interventions, including government support, capacity building, and digital infrastructure investment, are essential for the successful implementation of digital health systems in real-world settings for antibiotic stewardship.

## Data Availability

The original contributions presented in the study are included in the article/Supplementary Material, further inquiries can be directed to the corresponding author.
