# Supplementary material for: Antibiotic stewardship through clinical data digitization: perceived opportunities and obstructions by medical doctors from semi-urban setting in central India
Source: Front Digit Health. 2025 Sep 10;7:1652086. doi: 10.3389/fdgth.2025.1652086 (PMC12459205; doi:10.3389/fdgth.2025.1652086)
Supplement: Supplementary file 1 [file Datasheet1.docx]

**INTERVIEW GUIDE**

***“Antibiotic Stewardship through clinical data digitization: A qualitative study on opportunities and challenges by medical doctors from semi-urban setting in Central India”***

**Participant Information (details will be anonymized during data entry procedure)**

Name/ID (confidential)

Age: Gender: Years of medical practice:

Practice setting: (Private / Government / Both)

Specialization (if any):

Current designation:

SECTION 1: Introduction & Background

Purpose: To build relationship and understanding with the participant’s regarding his background and clinical setting.

Q1. Can you please tell me something about what kind of patients do you usually see?

Probes: What are the common infections you treat?

How often do you prescribe antibiotics?

Q2. What is do you know about antibiotic resistance and its effect in your practice?
Probes: How do you decide which antibiotic to use for a patient?

Q3. What do you think for the problem or the cause of antibiotic resistance in your region?

Probe: Are there system-level or patient-level factors you can identify?

SECTION 2: Clinical data documentation procedure

Purpose: To identify and understand the current record-keeping and data use.

Q5. How do you maintain clinical records for your patients in routine?
Probes: Is it paper-based, digital, or both?

Who maintains these records?

Q6. When and how many times need to follow patients’ previous prescriptions or patient history to antibiotic prescription?

Do you find it easy or difficult to retrieve past patient history?

Probe: Do you think access to past data is necessary for prescription may be antibiotic?

SECTION 3: Perceptions of digitization and electronic health records

Purpose: To assess understanding and attitude towards digital health records.

Q7. What do you understand by clinical data digitization or electronic health records?
Probes: Have you ever seen or used a digital record-keeping system?

What is your opinion on improving of patients care of electronic data of patient is there?

Q8. Do you think digitization can influence the antibiotic prescription practices?
Probes: Could it help reduce over-prescription or improve documentation?

Can it support evidence-based decisions or alerts for resistance?

SECTION 4: Potential of digitization in reducing antibiotic resistance

Purpose: To explore perceptions linking digitization to antibiotic resistance mitigation.

Q9. Do you think digitizing clinical data could play a role in antibiotic stewardship? Why or why not?
Probes: Would it help in identifying patterns of resistance in our area?

How can it assist in monitoring antibiotic use?

Q10. Can you suggest any system or software that can help you use antibiotics more judiciously?
Probes: Prescription audit system or software etc..

Antibiotic decision-support systems…

SECTION 5: Barriers and challenges

Purpose: To identify any barrier such as infrastructure, personal, or system barriers

Q11. What challenges do you see in implementing clinical data digitization in our setting?
Probes: Technology availability….computers, internet

Training and time constraints..

Patient load or anything else….

Q12. What do you think about patient data protection measures?

What is confidence on the digital system in this matter…………..

SECTION 6: Enablers and Recommendations

Purpose: for exploring the practical possibilities and policy suggestions

Q13. What kind of facilities or support would you need to use digitized clinical records in your daily practice?
Probes: Training or technical assistance……

Incentives or policy support…

Any other……

Q14. In your opinion what is role of government or local health authorities in digitization and controlling antibiotic resistance?
Probes: guidelines or mandatory systems…

Anything else…..

SECTION 7: Closing

Q15. Do you wish share anything more related to your experience with antibiotic resistance or digitization of clinical data?

**Notes for Interviewer**

Convey thank to participant for providing the time and consent for the interview

Ensure that interviews should be conducted in a quiet, private setting

Use audio recording with participant consent

Build confidentiality with the participant before starting

Adapt language as per the participant’s comfort (e.g., switching between English and Hindi).

Allow participants pause and silence in-between the communication to understand and respond
