# Supplementary material for: Antibiotic stewardship through clinical data digitization: perceived opportunities and obstructions by medical doctors from semi-urban setting in central India
Source: Front Digit Health. 2025 Sep 10;7:1652086. doi: 10.3389/fdgth.2025.1652086 (PMC12459205; doi:10.3389/fdgth.2025.1652086)
Supplement: Supplementary file 2 [file Table1.docx]

### **Audit Trail**

This audit trail documents the research process followed in the qualitative study, ensuring transparency and methodological rigour throughout the thematic analysis. The study followed Braun and Clarke’s (2006) six-phase approach to thematic analysis and adopted an inductive, data-driven coding process.

| **Stage** | **Description of Activities** |
| --- | --- |
| **1. Data Collection** | Twenty clinicians were interviewed using semi-structured interview guides. Two interviews were incomplete due to time constraints and excluded from analysis. Eighteen interviews were audio-recorded, transcribed verbatim, and anonymized. |
| **2. Familiarisation with Data** | All transcripts were read multiple times by two members of the research team to immerse in the data. Preliminary notes and observations were documented. |
| **3. Generation of Initial Codes** | An inductive coding approach was applied manually using Microsoft Excel. Initial codes were generated line-by-line across all transcripts, without using a pre-existing coding framework. Examples of early codes included: "loss of prescription", "repeat antibiotic", "no internet", "policy mandate", "real-time access", "lack of training". |
| **4. Searching for Themes** | Codes were grouped into meaningful categories and patterns. Similar codes were clustered and reviewed iteratively to identify potential themes and subthemes. A codebook was developed collaboratively among researchers. |
| **5. Reviewing Themes** | Themes and subthemes were reviewed in relation to the coded extracts and full dataset to ensure internal coherence and external heterogeneity. Codes and themes were refined in group discussions with the full research team. |
| **6. Defining and Naming Themes** | Final themes and subthemes were defined and refined to accurately reflect participant perspectives. Each theme was supported with illustrative quotations. A thematic map was developed to guide narrative construction. The four main themes that emerged were: (1) Digitization enhances accountability and continuity of care, (2) Potential for localized real-time antimicrobial resistance surveillance, (3) Infrastructural and technological barriers, and (4) Necessity of government support and capacity building. |
| **7. Producing the Report** | The final manuscript integrates participant quotes to substantiate analytical interpretations. Themes are aligned with categories and codes (Table 2) to ensure traceability from raw data to reported findings. |
| **8. Data Management** | Transcripts and codes were stored securely and managed using password-protected spreadsheets. Coding and theme development was manually conducted. |
